# Graphene Nanofiller Type Matters: Comparative Analysis of Static and Fatigue Delamination Resistance in Modified Carbon Fiber Composites

**DOI:** 10.3390/polym17243299

**Published:** 2025-12-12

**Authors:** Konstantina Zafeiropoulou, Christina Kostagiannakopoulou, George Sotiriadis, Vassilis Kostopoulos

**Affiliations:** 1 Department of Mechanical Engineering & Aeronautics, University Campus Patras, GR-26504 Rio, Achaia, Greece; k_zafeiropo@ac.upatras.gr (K.Z.); kostagia@upatras.gr (C.K.); sotiriad@upatras.gr (G.S.); 2 Foundation of Research and Technology, Institute of Chemical Engineering Sciences (FORTH/ICE-HT), Stadiou Str., GR-26504 Rio, Achaia, Greece

**Keywords:** fracture properties, mode I fatigue, composite laminates, graphene nanoplatelets, SEM

## Abstract

Delamination remains a critical failure mode in carbon fiber-reinforced polymer (CFRP) composites, particularly under cyclic loading in aerospace and automotive applications. This study explores whether nanoscale reinforcement with graphene-based materials can enhance delamination resistance and identifies the most effective nanofiller type. Two distinct graphene nanospecies—reduced graphene oxide (rGO) and carboxyl-functionalized graphene nanoplatelets (HDPlas)—were incorporated at 0.5 wt% into CFRP laminates and tested under static and fatigue mode I loading using double cantilever beam (DCB) tests. Both nanofillers enhanced interlaminar fracture toughness compared to the neat composite: rGO improved the energy release rate by 36%, while HDPlas achieved a remarkable 67% enhancement. Fatigue testing showed even stronger effects, with the fatigue threshold energy release rate rising by 24% for rGO and 67% for HDPlas, leading to a fivefold increase in fatigue life for HDPlas-modified laminates. A compliance calibration method enabled continuous monitoring of crack growth over one million cycles. Fractography analysis using scanning electron microscopy revealed that both nanofillers activated crack bifurcation, enhancing energy dissipation. However, the HDPlas system further exhibited extensive nanoparticle pull-out, creating a more tortuous crack path and superior resistance to crack initiation and growth under cyclic loading.

## 1. Introduction

CFRP composite materials are extensively deployed across real-world engineering fields such as automotives, aeronautics, wind energy, marine applications, and sports equipment, thanks to their significant advantages over alternative materials. However, the brittle nature of the polymer matrix and the fiber–matrix interface can lead to potential failure due to delamination. Delamination represents a major structural integrity issue in laminated composites because it can limit the service life of the composite elements and may lead to catastrophic failure [1].

For this reason, many studies have been focused on toughness improvement by adding nanoparticles such as carbon nanotubes and graphene nanospecies to the matrix material [2,3,4,5,6,7]. Among the nanoreinforcing materials, graphene has attracted significant scientific interest due to its superior mechanical, thermal and electrical properties, and its additional fracture mechanisms, such as crack bifurcation and layer separation. Many publications reporting quasi-static monotonic loading results for both fracture modes are available. Kumar et al. [8] investigated the mode I fracture properties of epoxy/carbon fiber unidirectional laminate with and without dispersed hydrogen-passivated nanographene platelets (HP-NGPs). A nearly 100% enhancement in crack-propagation resistance was recorded for the system reinforced with 0.5 wt% NGP relative to the neat specimens. Soyugüzel et al. [9] carried out a study on the toughening effect of nitrogen-doped reduced graphene oxide particles (ND-RGOP) in mode I and mode II delamination of carbon fiber/epoxy laminates. In mode I, the incorporation of 0.8 wt% ND-RGOP led to significant improvements in both delamination initiation and propagation toughness, by 126% and 119%, respectively. Beyond the mode I toughness enhancements, ND-RGOP addition also improved mode II fracture resistance. The maximum mode II delamination toughness was achieved with 0.4 wt% ND-RGOP, exhibiting a 45.6% increase relative to neat samples. Tareq et al. [10] also added graphene nanoplatelets (GNPs) to CFRPs. The critical mode I interlaminar fracture toughness of the GNP-reinforced composites was enhanced by 40% compared to control samples.

During the structural applications, CFRPs experience a range of loading conditions, including cyclic loads, which also affect their performance and lifespan significantly. The desire and need to improve the fatigue behavior of CFRPs has steadily intensified and has led to research efforts evaluating the delamination onset and growth of CFRP laminates under cyclic fatigue load conditions [11,12,13,14,15,16,17]. Most research has been focused either on the fatigue mechanical properties of modified CFRPs [18,19], or on the fatigue fracture characteristics of reinforced nanocomposites [20]. With a glance at the open literature, very limited studies are available about the interlaminar fatigue performance of CFRP composites modified with fillers, because of the complexity and time consumption of these tests. Romhány et al. [21] studied the influence of carbon nanotubes on the mode I interlaminar fatigue behavior of CFRP composites. The authors proposed a localization methodology for monitoring the interlaminar fatigue crack front based on acoustic emission (AE) measurements. Experimental results revealed that the addition of carbon nanotubes led to a 69% reduction in crack propagation rate relative to composites without nanotube reinforcement. Furthermore, a notable enhancement in fatigue life was observed, as the nanotube-modified composite endured approximately 3.8 times more loading cycles to failure than the unmodified matrix composite. Kotrotsos et al. [22] prepared 1 wt% GNP-modified unidirectional (UD) CFRPs with Bis-maleimide (BMI) resin. The enhanced resin was selectively incorporated into the mid-thickness region of the high-performance laminates through a solution electrospinning process (SEP). Experimental analysis revealed that BMI-modified CFRPs demonstrated the highest resistance to delamination under mode I fatigue loading conditions, whereas the laminates modified with BMI and GNP presented superior performance under mode II fatigue loading conditions.

The aim of this study is to investigate the effect of the graphene nanospecies on the mode I static and fatigue delamination growth of CFRPs and identify the various fatigue improvement mechanisms contributed by nanofillers.

## 2. Methodology

Fracture mechanics-based delamination growth models are required to predict the damage tolerance of composite laminates. Whereas there are standardized test methods for measuring the static fracture toughness (*G_IC_*) [23] and the fatigue threshold value on delamination onset (*G_Ith_*) [24] under mode I loading, there is currently no standard for determining the delamination growth rate in fatigue load conditions. Recently, Paris and O’Brien established an empirical relation between the *da/dN* and the strain energy release rate (*G*) to characterize the stable delamination growth:

(1) $$\frac{d\alpha }{dN}=C\cdot {f(G)}^{m}$$

where *C* and *m* are material constants. Several versions of Paris’ law have been reported in the literature [15]. Both *f*(*G*) = *G_max_* and *f*(*G*) = Δ*G* have been successfully correlated with crack propagation by many researchers.

**Figure 1:**
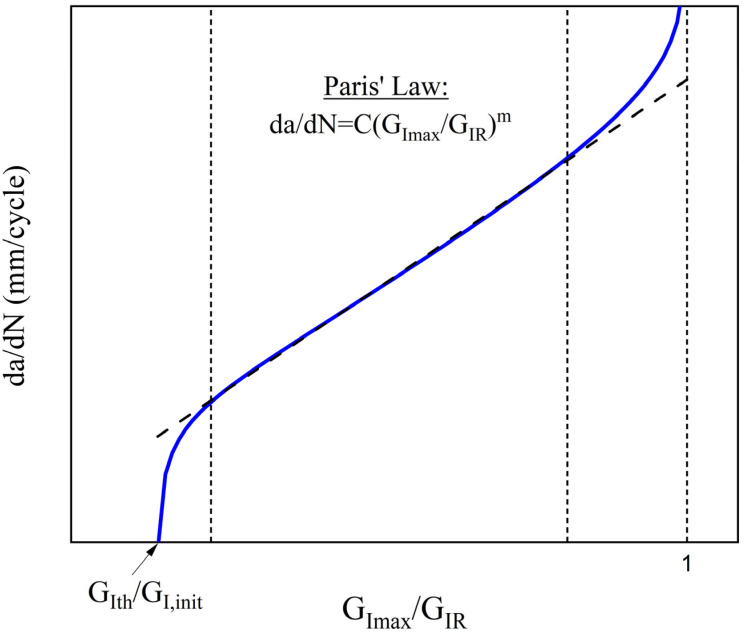
Schematic curve of *da/dN* versus *G_Imax_/G_IR_*. The dashed line represents the linear Paris’ law region used to determine the parameters *C* and *m*.

Because the DCB specimens are UD, the nesting of fibers between adjacent plies can occur during delamination growth, resulting in fiber-bridging at the delaminating interface, which increases the apparent fracture toughness [25] and affects the resulting Paris’ law. Therefore, in order to correct the influence of fiber-bridging, many researchers adopted the normalization by *G_IR_* (Figure 1). *G_IR_*(*α*) is a delamination resistance curve (R-curve), which comes from the quasi-static loading and reduces the spread of delamination growth data during the fatigue loading.

Another issue that arises is the computation of delamination length during the fatigue tests. In most cases, the test is interrupted at predetermined cycle intervals so that the delamination length can be measured using a traveling microscope [13,14,22]. This procedure is time-consuming and requires the presence of the operator so as to stop the test and record the crack tip. For this reason, compliance calibration (CC) is presented in some studies [26]. CC is an experimental method, which was first introduced for fatigue testing in ENF specimens [27]. The benefit of this procedure is that the fatigue test can be performed without any interruption for visual inspection of delamination length and can produce a large number of delamination growth data points. The relationship between the compliance and the delamination length of the specimen is determined through the equation:

(2) $$C=m\cdot \alpha^{3}+A$$

where *α* is the delamination length and *m*, and *A* are material-specific fitted parameters obtained from the compliance calibration procedure.

## 3. Material Properties and Specimens’ Preparation

### 3.1. Material

**Figure 2:**
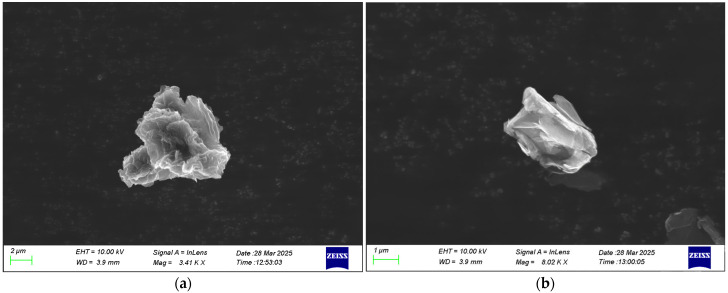
SEM image of (**a**) rGOs and (**b**) HDPlas nanofillers, respectively.

All materials utilized in the present study are commercially available. The matrix material used was a two-component epoxy system, supplied by Fibermax Composites (Agria Volou, Greece) and designed for in-house fabric pre-impregnation. This system consists of the thermosetting epoxy resin R244 and the hardener H33, which are typically mixed in the ratio 100:21 by weight, according to the manufacturer’s recommendation. The main reinforcement phase was a unidirectional carbon fabric PX35, 500 mm wide, provided by R&G Composite Materials (Waldenbuch, Germany) with an areal density of 200 g/m^2^ and a 75 dtex PET binding yarn on one side. As regards the nanofillers, two different kinds of graphene nanospecies were used for the current work: reduced graphene oxide (rGO) supplied by Nanoinnova Technologies (Toledo, Spain) and graphene nanoplatelets that were exfoliated through plasma and then modified with carboxyl (COOH-HDPlas_GNPs) obtained from Graphene Laboratories (Ronkonkoma, NY, USA). The former has a surface area of 103.2 m^2^/g, while the latter ranged in size from 8 to 13 μm. The latter nanofiller has a bulk density of 215 kg/m^3^ with an average diameter of 3.8 μm. The platelets have a thickness of less than 50 nm and a typical surface area of 20 m^2^/g, according to the product data sheet. The weight fraction of both nanofillers was fixed at 0.5% in order to avoid agglomerations, which can be found at higher weight contents [28]. Figure 2 depicts SEM images for the as-received graphene nanofillers in order to obtain an impression of the morphology of each filler type used in this study. A 3D-networked nanostructure of irregular shape can be observed in the SEM image of rGO, while in the case of HDPlas, some platelets are formed in the main filler volume.

### 3.2. Development of Laminates

**Figure 3:**
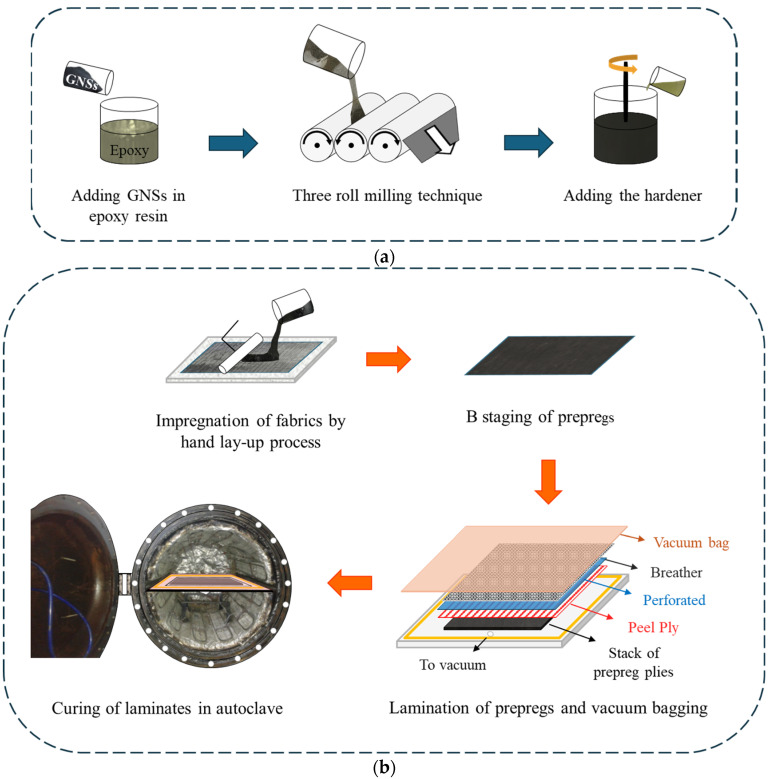
Schematic illustration of (**a**) the nanomodified matrix preparation and (**b**) the CFRP fabrication process.

Composite laminates were manufactured, as illustrated analytically in Figure 3. For the preparation of the nanomodified matrix, a three-roll milling process was followed, as described in previous work [29] that secures the homogeneous distribution of the nanofillers. The prepared mixtures were used for the impregnation of the UD fabrics with a view to produce pre-impregnated plies. The prepreg technique followed was the hand lay-up process. A neat resin system was additionally utilized as a matrix to establish the reference material properties. Impregnation of the fabric was conducted at room temperature conditions, after which the prepregs were subjected to a 48 h b-staging process at the same conditions, as recommended by the manufacturer. Three 16-ply laminates (neat, rGO, HDPlas) were produced using a vacuum bagging technique, with a polytetrafluoroethylene (PTFE) film of 62.5 mm length inserted at the mid-plane to create the initial delaminated region. The plates were cured in an autoclave oven following the manufacturer’s guidelines at 120 °C for 1 h under a pressure of 6 bar. The temperature and pressure were controlled by an, in-house developed, integrated software system (LabView) connected to a computer. The resulting laminates were approximately 305 mm long and 260 mm wide, with an average thickness of 3.36 mm and a fiber volume fraction *vf* between 50 and 52%.

**Figure 4:**
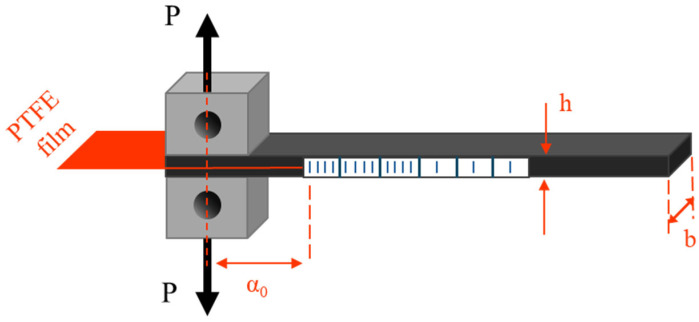
Specimen configuration for fracture tests.

Double cantilever beam (DCB) tests were carried out for the determination of the opening mode I interlaminar fracture toughness (*G_IC_*). For this reason, DCB specimens were cut from the manufactured laminates, 250 mm long and 25 mm wide, with an effective initial crack length of 50 mm measured from the loading line (the above-mentioned 62.5 mm refers to the PTFE film length inserted during manufacturing, while the 50 mm effective initial crack length accounts for the crack measurement from the loading line). Five specimens were tested from each type of material system and were designed according to the ASTM D 5528 standard [23]. Loading blocks and specimens were ground, polished and subsequently cleaned with acetone prior to bonding. Aluminum loading blocks were then attached to the notched regions of the beam, as shown in Figure 4. One side of each specimen was painted white to facilitate clear visualization of the crack propagation.

## 4. Methods and Results

### 4.1. Mode I Quasi-Static Test

The fracture test was performed in a servo-hydraulic test machine (Instron 8872 supplied by Analytical Instruments S.A., Athens, Greece) using a 1 kN load cell. The loading blocks of the specimen were supported through pins in a loading yoke, which was mounted in the hydraulic grips of the load frame. The tests were carried out by displacement control loading at 5 mm/min. The applied loading and the opening displacement of each specimen were recorded during the test, while the crack-tip location was periodically measured and documented together with the corresponding load and displacement for each recorded crack propagation. The crack advance was also measured at regular intervals by the opposite side of the samples and no significant differences were identified.

**Figure 5:**
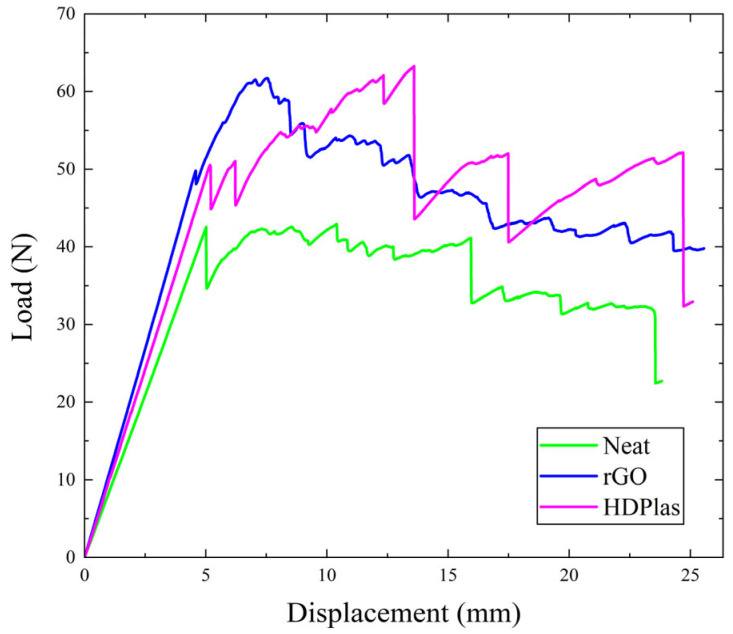
Representative load–displacement response for representative specimen from each material (neat, rGO and HDPlas).

**Figure 6:**
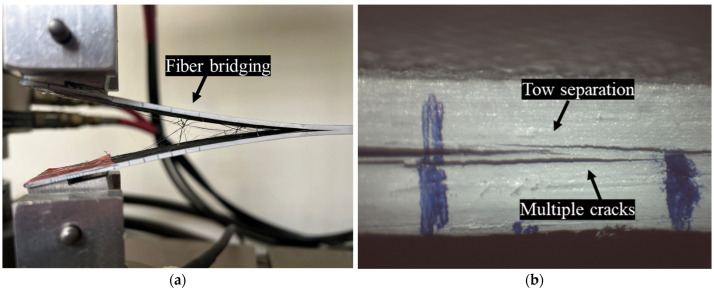
Edge failure modes: (**a**) fiber bridging and (**b**) tow splitting and interlayer separation observed in HDPlas-doped specimens.

The load-versus-displacement curves for the three composites are depicted in Figure 5. This response exhibits the typical behavior of brittle matrix composite laminates. Examination of the specimen edge revealed predominant fiber bridging, while for the HDPlas-doped specimens, additional failure mechanisms, including tow splitting and interlayer separation, were also observed (Figure 6).

**Figure 7:**
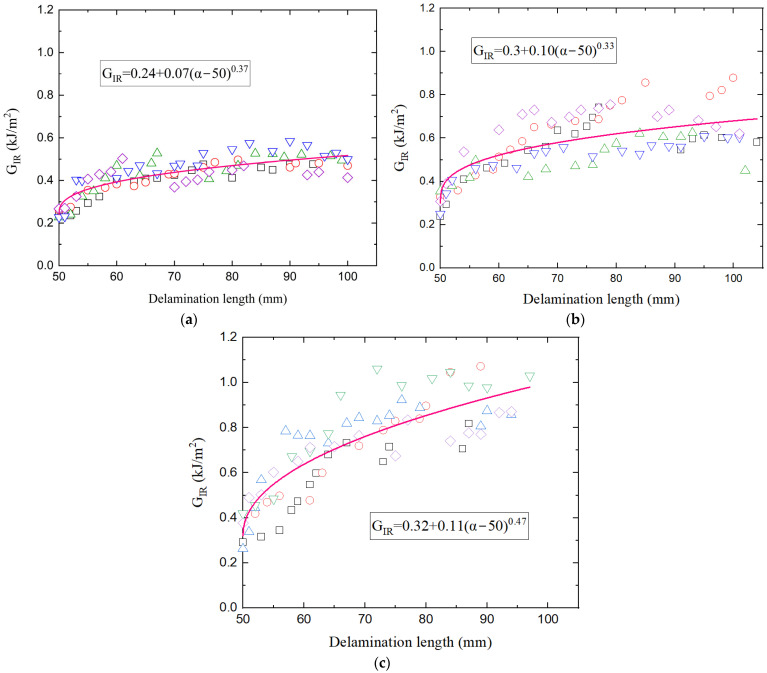
Energy release rate versus delamination length for (**a**) neat, (**b**) rGO, and (**c**) HDPlas composites. The different data shapes correspond to the five individual specimens tested for each material type.

The energy release rate *G_I_* was calculated from the Modified Beam Theory (MBT) method according to Equation (3):

(3) $$G_{I}\;=\;\frac{3P\delta }{2b(a+\left|\Delta \right|)}$$

where *P* = load, *δ* = applied displacement, *b* = specimen width and *α* = delamination length. The parameter Δ serves as a correction factor for the delamination length to account for the non-ideal built-in boundary conditions of the DCB specimen. The Δ was obtained by the specimen compliance and the delamination length data [23]. The initial fracture toughness *G_I,init_* was determined by the point at which delamination growth was observed on the crack tip of the specimen. The results of *G_I,init_* as well as of the average *G_I_* are depicted in Table 1. It is clear that both nanofillers enhanced the interlaminar fracture toughness of the neat CFRP. Specifically, rGO performed an increase of 25% in *G_I,init_* and 36% in *G_I_*, while the HDPlas provided even greater improvements of 33% and 67%, respectively. Energy release rate *G_IR_* was also expressed as a function of *G_I,init_* and the delamination extension (α − 50) and plotted against delamination length α as shown in Figure 7. A power-law equation was fitted to the data and the resulting resistance equation is shown on the diagram for each composite material. Since the diagrams share a comparable scale, it is clear that both nanoreinforced systems exhibited higher *G_IR_* values compared to the neat composite, indicating improved resistance to crack propagation. Among them, the HDPlas composite consistently demonstrated the highest *G_IR_* values across the entire delamination range. The curves show a gradual increase in *G_IR_* with delamination length, suggesting a stable crack growth regime. The fitted power-law relationship, expressed as *G_IR_ = G_I,init_ + A*(*α* − *α*_0_)*^n^*, provides insight into the evolution of fracture resistance with crack extension. The exponent n characterizes the sensitivity of *G_IR_* to delamination length-lower values of neat and rGO composites correspond to a more gradual increase in resistance, whereas higher values (n = 0.47) of HDPlas composites indicate a stronger dependence of *G_IR_* on crack growth, resulting in a steeper curve. This behavior reflects the progressive activation of energy-dissipating toughening mechanisms as the crack advances, leading to enhanced fracture resistance and overall toughness of the material.

### 4.2. Compliance Calibration Method

**Figure 8:**
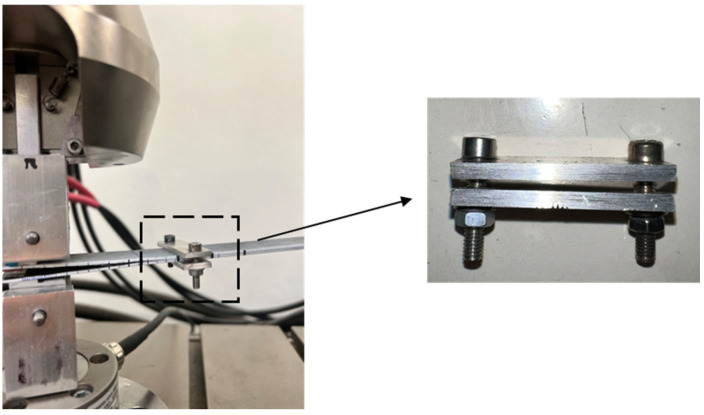
Compliance calibration test with the use of a clamp.

**Figure 9:**
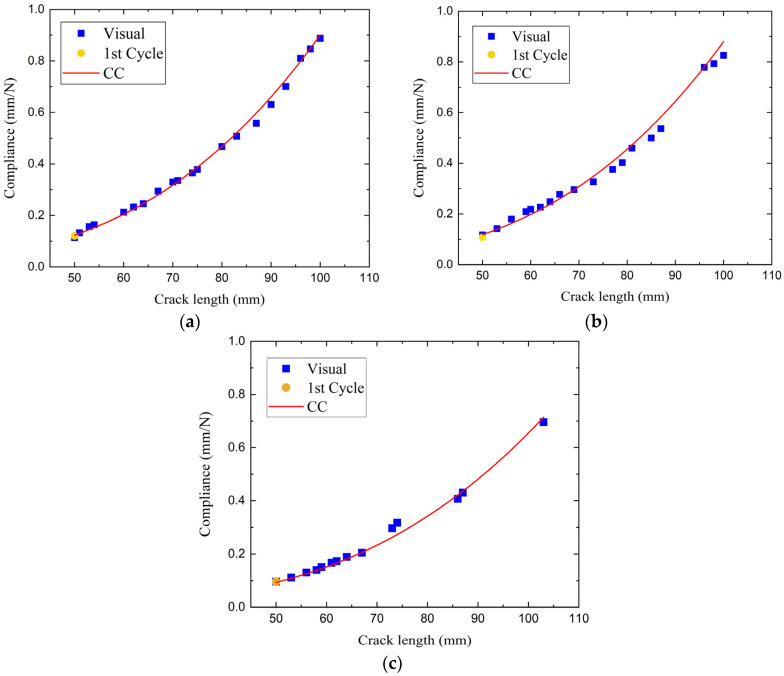
Mode I compliance calibration results for (**a**) neat, (**b**) rGO, and (**c**) HDPlas composites. The square dot is the compliance calculated from the visual observation data during the quasi-static test.

Compliance calibration (CC) was carried out after completion of the fatigue cycling, with the loading blocks still attached to the specimens. To ensure precise delamination length, a clamp was mounted on the specimens, as illustrated in Figure 8. The bolts were tightened sufficiently to hold the two halves of the specimens in contact and prevent the crack opening within the clamped zone. The specimens were loaded at a test speed of 1 mm/min up to a displacement value below that recorded in the quasi-static test, followed by unloading. The compliance was calculated from the slope of the linear part of the force–displacement curve. The procedure was repeated for a series of clamp positions corresponding to different delamination lengths (50 mm, 70 mm, 90 mm, 110 mm) and the relation between the delamination length and the compliance was evaluated by the function in Equation (2). This procedure was applied to all DCB specimens. The experimental data, obtained from the quasi-static tests, fitted very well with the relation defined by Equation (2), with the coefficient of determination R^2^ between 0.97441 and 0.9976. The compliance measured in the first fatigue loading cycle, when the delamination length is known, was in agreement with the results of CC too. Three representative graphs of delamination length vs. compliance, corresponding to three specimens from each material system, are presented in Figure 9.

### 4.3. Fatigue Onset Life

**Figure 10:**
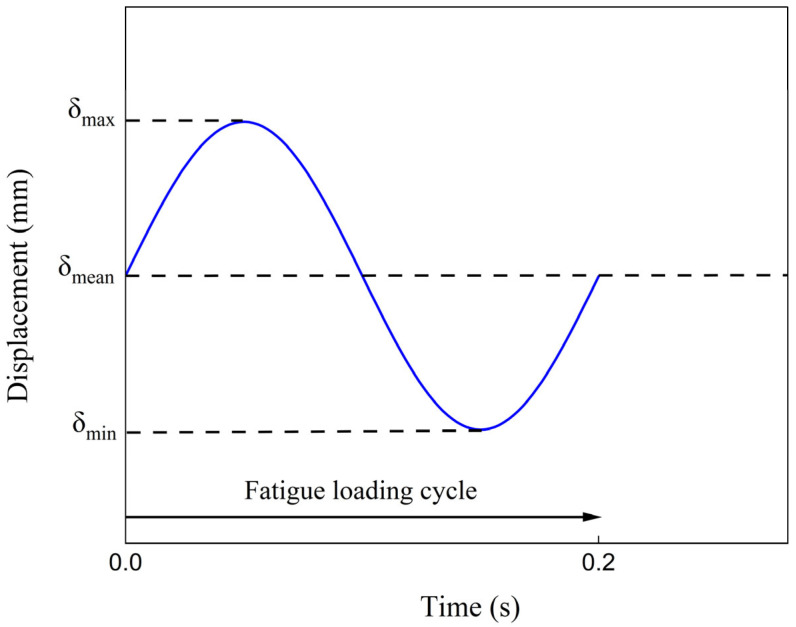
Applied sinusoidal fatigue loading cycle with a cyclic frequency of 5 Hz.

Fatigue delamination onset life tests were performed in accordance with ASTM D 6115 [24] to evaluate the threshold energy release rate value *G_Ith_*. Five specimens—200 mm long, 25 mm wide, with initial crack length of 50 mm—were tested from each material system under fatigue conditions for the onset tests. The fatigue tests were conducted under constant amplitude of displacement at a cyclic frequency of 5 Hz (Figure 10). The low frequency (5 Hz) used concludes to minimum temperature change in specimens during fatigue (max 0.5 °C). The displacement ratio, *R = δ_Imin_/δ_Imax_*, was equal to 0.1. Values of *δ_Imax_* were obtained from the equation:

(4) $$\frac{{\delta_{Imax}}^{2}}{{\delta_{I,init}}^{2}}\;=\;\frac{G_{Imax}}{G_{I,init}}$$

where *δ_I,init_* is the displacement when the delamination starts to grow in the quasi-static test, to apply G-levels of 50% and 80%. The load, the displacement and the number of cycles were stored by the machine software in a text file for subsequent post-test evaluation.

**Figure 11:**
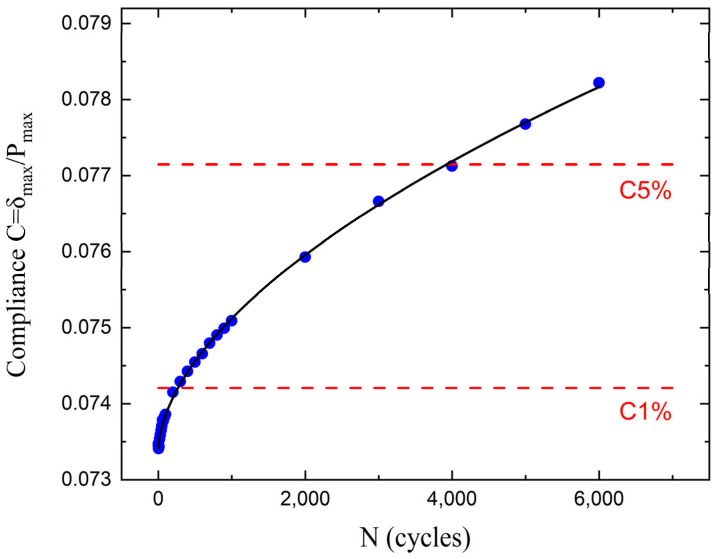
Compliance versus number of cycles in fatigue onset life test with both 1% and 5% increase in compliance.

**Figure 12:**
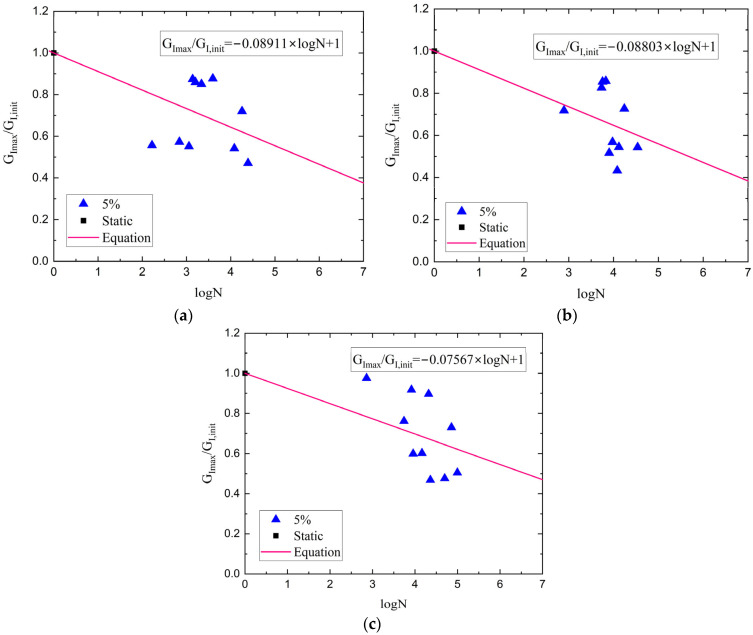
Variation in onset fatigue life with G_Imax_/G_I,init_ for G-levels 50% and 80% for (**a**) neat, (**b**) rGO, and (**c**) HDPlas composites.

The ASTM D 6115 standard recommends two criteria for calculating delamination onset life: a 1% and a 5% increase in compliance relative to the compliance at the first cycle (*N* = 1) (Figure 11). Table 2 summarizes the average onset lives for the composites at both 50% and 80% *G_Imax_*/*G_I,init_* ratios. Because the 1% criterion is too conservative, the 5% compliance change criterion was selected to establish the critical values. Fatigue life evaluation revealed that the incorporation of nanofillers substantially improved the durability of the composites under cyclic loading. Specifically, at 50% of G-level, the rGO led to an approximate twofold increase in fatigue onset life, while the HDPlas provided an even more pronounced effect, extending fatigue onset life by nearly five times, compared to the neat composite. A similar trend was observed at the higher loading G-level of 80%, where the fatigue onset life was still enhanced by 33% for the rGO composite, while HDPlas continued to exhibit a substantial enhancement of nearly four times. Figure 12 shows the curve of *G_Imax_*/*G_I,init_* versus log*N* data for 5% criteria. O’Brien [30] proposed a linear relationship between *G_Imax_* and log*N* data for 10^0^ < *N* < 10^6^, and then he calculated the *G_Ith_* value from the fitted equation at *N* = 10^6^. For the present study, an identical linear equation was used to fit the data, while the threshold energy release rate *G_Ith_* was determined at *N* = 10^7^ (see Table 2). This extrapolation beyond the tested range assumes that the linear relationship remains valid, though this introduces uncertainty that should be considered when applying these threshold values. This means that when the applied energy release rate is below the calculated value for each material, then the delamination will not propagate up to 10 million cycles. It is also clear that the incorporation of the graphene nanospecies in a CFRP structure increased the delamination propagation threshold, with improvements of 24% and 67% for the rGO and HDPlas fillers, respectively.

### 4.4. Fatigue Delamination Growth Rate

**Figure 13:**
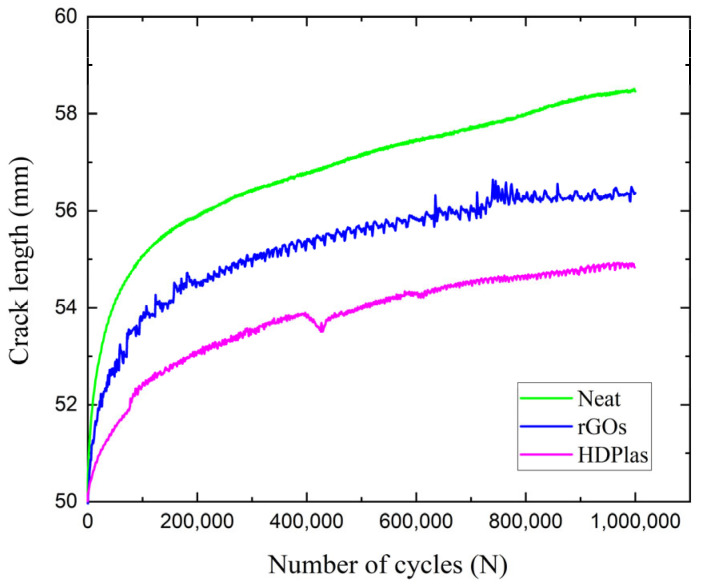
Representative crack length versus the number of cycles for neat, rGO and HDPlas composites, under mode I fatigue loading conditions.

The same set-up with fatigue onset life tests was also utilized in the fatigue propagation tests. The applied *δ_Imax_* corresponded to the *G_Imax_*/*G_I,init_* ratio of 80%. All specimens were already precracked from the onset life tests. The fatigue tests were run for a predetermined number of cycles of 10^6^. During the delamination growth rate tests, the load and the displacement were recorded, while the crack length was obtained from each CC curve material. Figure 13 depicts the relation between the propagation of the crack length and the number of cycles during the fatigue delamination growth for all materials. According to this diagram, it is clear that the HDPlas composite exhibited the highest resistance to delamination as the crack propagated only 4.8 mm (from 50 mm to 54.8 mm). On the contrary, the neat CFRP exhibited the worst delamination resistance as the crack length extended by 8.5 mm (from 50 mm to 58.5 mm), while the rGO composite presented a slightly lower crack extension of 6.3 mm (from 50 mm to 56.3 mm) against the neat one.

**Figure 14:**
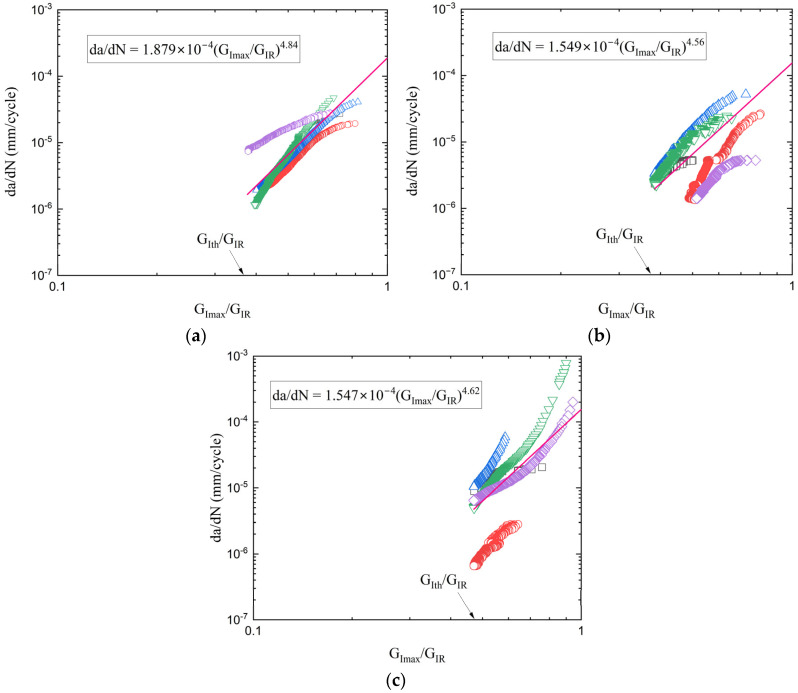
Delamination growth rate results for (**a**) neat, (**b**) rGO, and (**c**) HDPlas composites. The different data colors correspond to the five individual specimens tested for each material type, while the magenta line is the fitted curve of the corresponding data.

From the results, *da/dN*, *G_Imax_*, and *G_IR_* were also obtained. The *G_Imax_* was calculated from the equation:

(5) $$G_{Imax}\;=\;\frac{3P_{Imax}\delta_{Imax}}{2b(a+\left|\Delta \right|)}$$

where |Δ| was calculated from the CC method too. The values of *da/dN* were obtained by differentiating the crack-growth curves shown in Figure 13 for each material system. Results of the delamination growth rate tests for the three composites are shown in Figure 14. The experimental data and the corresponding equations were constrained within the limits *G_Imax_* = *G_Ith_* and *G_Imax_*/*G_IR_* = 1. The threshold value *G_Ith_* is proportional to *G_I,init_*, whereas the upper limit *G_Imax_*/*G_IR_* = 1 arises from the condition that *G_Imax_* = *G_IR_* corresponds to an infinite or unstable delamination growth rate. The propagation data were fitted by the Paris’ law form:

(6) $$\frac{d\alpha }{dN}\;=\;C{\left(\frac{G_{Imax}(\alpha )}{G_{IR}(\alpha )}\right)}^{m}$$

where *m* has no unit and *C* is the speed of delamination growth when *G_Imax_* = *G_IR_* at fast fracture. Based on the fitted curves derived from the propagation data points, the Paris’ law coefficients were calculated to be *m* = 4.84 and *C* = 1.88 × 10^−4^ for the neat CFRP, *m* = 4.56 and *C* = 1.55 × 10^−4^ for the rGO composite, and *m* = 4.62 and *C* = 1.54 × 10^−4^ for the HDPlas composite.

The Paris law parameter *C* represents the crack growth rate at the instability condition (*G_Imax_ = G_IR_*) and shows similar values for all three materials (1.54–1.88 × 10^−4^ mm/cycle). This indicates that once the critical condition is reached, all materials fail at comparable rates. However, the superior fatigue performance of HDPlas stems primarily from its 67% higher threshold energy release rate (*G_Ith_*), which allows the material to sustain significantly higher loads before approaching the instability regime. The similar exponent *m* values (4.62–4.84) suggest that the acceleration of crack growth toward instability follows comparable trends, with the initial threshold being the dominant factor in determining fatigue life.

The noticeable scatter observed in the fatigue data can be attributed to the inherent variability of fatigue damage processes and the high sensitivity of crack propagation to local microstructural features. In composite materials, factors such as fiber–matrix interfacial strength, voids, resin-rich areas, and local stress concentrations introduce significant heterogeneity in the stress field, leading to specimen-to-specimen variations in fatigue life [31,32]. Additionally, differences in the dispersion and orientation of nanofillers, as well as slight inconsistencies in manufacturing, can further influence the initiation and propagation of microcracks under cyclic loading. These combined effects result in a stochastic fatigue response, where small microstructural variations produce large fluctuations in the measured fatigue crack growth rates or lifetime.

### 4.5. Fractography Analysis of Fracture Surfaces in Mode I Static and Fatigue Tests

**Figure 15:**
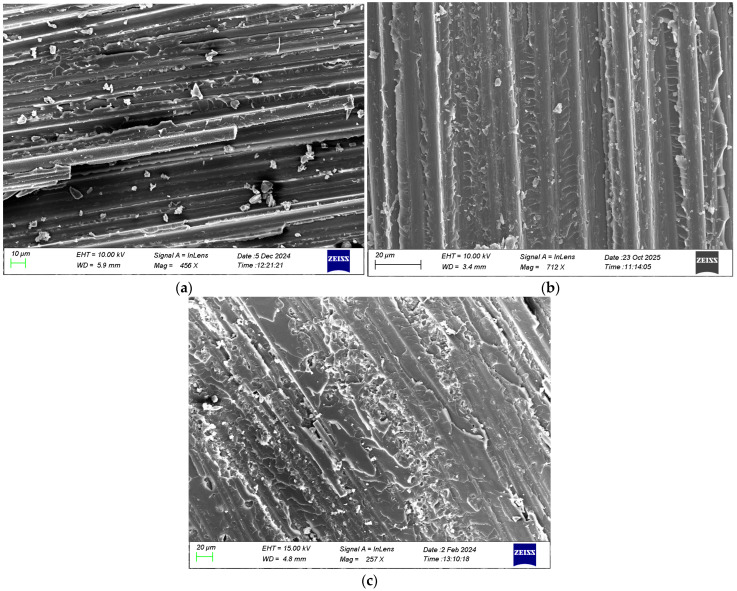
Representative SEM images of fracture surfaces at low magnification in static test of (**a**) neat, (**b**) rGO, and (**c**) HDPlas composites.

A scanning electron microscopy (SEM) fractographic analysis was carried out on material samples extracted from the crack propagation region of specimens subjected to mode I monotonic and cyclic loading. The samples were gold-coated and detailed observations of the fracture surfaces were made at different magnifications. The fracture morphology under mode I monotonic loading in Figure 15 shows a smooth surface for the neat composite associated with the brittle nature of the epoxy material, while the nano-enhanced composites exhibit rougher surfaces due to the existence of graphene nanospecies.

**Figure 16:**
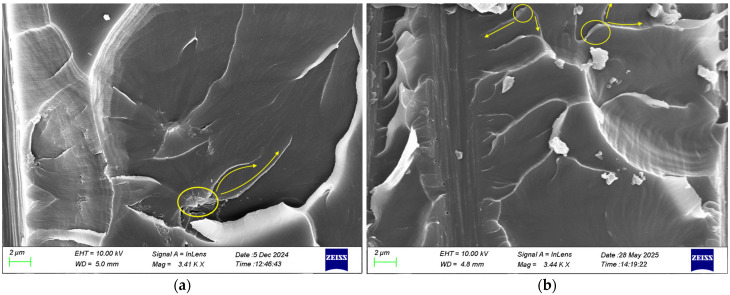
SEM images showing bifurcation of crack tip mechanism in (**a**) rGO and (**b**) HDPlas composites under static loading. The yellow circle highlights the main crack, while the arrows indicate the secondary crack branching.

**Figure 17:**
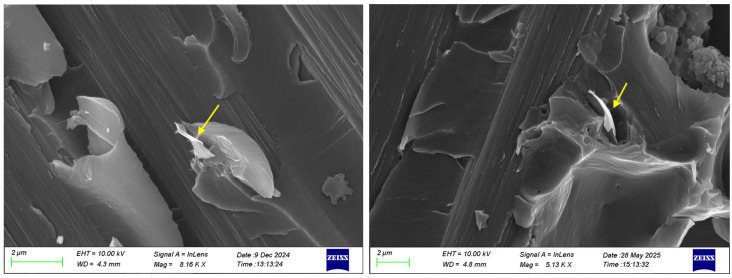
SEM images revealing the additional fracture mechanism of HDPlas, pull-out of fillers under static loading. The yellow arrow indicates a filler layer being pulled out from the matrix.

In nanomodified composite materials, the failure mechanism often exhibits complex features due to the interaction between the reinforcing nanoparticle and the polymer matrix. One phenomenon that is observed in graphene-based composites is crack bifurcation [33]. In Figure 16, the SEM images clearly demonstrate that the main crack splits into two secondary paths, indicated by the arrows, with both graphene nanoparticles incorporated, as circled in the images. This observation explains the delayed crack growth and the increased energy absorption capacity, which underline the improved damage tolerance of the nanomodified composites under the monotonic loading compared to the neat system. However, the comparative analysis between the two nanomodified systems highlighted distinct differences in their toughening mechanisms. In the case of the HDPlas composite, SEM observations revealed an additional pull-out mechanism (Figure 17), which is absent in the rGO composite. This pull-out process arises from interfacial debonding, followed by frictional sliding during HDPlas fillers extraction, contributing substantially to the overall energy absorption. From a fracture mechanics perspective, the activation of pull-out provides a gradual resistance to crack propagation, thereby increasing the effective strain energy release rate and delaying unstable crack growth.

**Figure 18:**
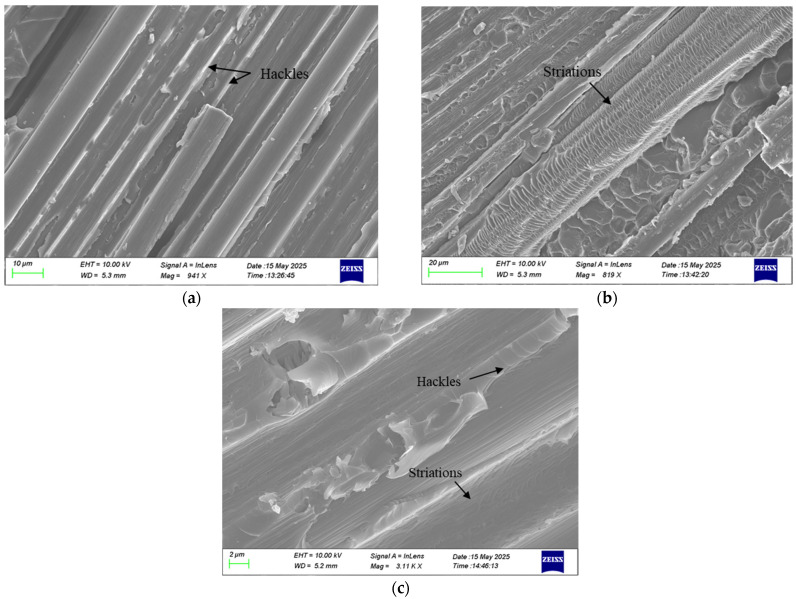
SEM images of fracture surfaces showing hackles and fatigue striations in the (**a**) neat, (**b**) rGO, and (**c**) HDPlas composites.

In both static and fatigue fracture surfaces, typical fracture features such as fiber breakage and imprints were observed. The main difference between these two crack propagation regions was the presence of hackles and striations in the fatigue zone. The observed hackles created teeth-like patterns in the matrix between adjacent fiber imprints (Figure 18). Hackles are created because of microcracks that develop ahead of the crack tip, within the plane experiencing the highest tensile stress. When the main crack interacts and merges with these microcracks, the characteristic hackle patterns are produced [34]. Fatigue striations were also identified within the fiber imprints on the fracture surfaces. Such striations arise from the incremental fiber–matrix debonding that occurs during a fatigue cycle. Figure 18 depicts typical striations on the fracture surface of samples.

**Figure 19:**
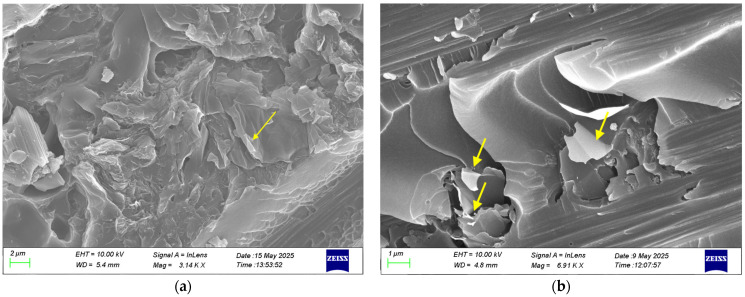
SEM images showing the pull-out mechanism in (**a**) rGO and (**b**) HDPlas composites under cyclic loading. The yellow arrow indicates a filler layer being pulled out from the matrix.

Under cyclic loading, both nanomodified composites exhibited evidence of nanoparticle pull-out, confirming the activation of this energy-dissipating mechanism during progressive crack growth. However, the extent of pull-out was found to differ significantly between the two materials. In the case of the rGO composite, only limited pull-out was observed (Figure 19a), resulting in a modest contribution to crack growth resistance and a correspondingly smaller improvement in energy absorption under fatigue. In contrast, the HDPlas composite displayed more extensive pull-out, with nanoparticles debonding along the crack path (Figure 19b), thereby promoting frictional sliding and progressive energy dissipation. This difference explains the superior fatigue performance of HDPlas filler, where the higher degree of pull-out contributed to delaying crack propagation and enhancing fatigue life.

## 5. Conclusions

This experimental investigation has shown that the incorporation of nanofillers into fiber-reinforced composites effectively enhanced mode I fracture resistance under both static and fatigue loading. The neat composite exhibited the lowest fracture energy, whereas the addition of rGO and HDPlas nanofillers led to marked increases of 36% and 67% in static toughness, and 24% and 67% in fatigue threshold energy release rate. The experimental compliance calibration method applied to the DCB specimens estimated delamination length values that were consistent with both visually recorded lengths and calculated effective delamination lengths. Its simplicity, reliability and low instrumentation requirements enable rapid estimation of delamination progression, making it suitable for engineering and industrial environments where the assessment of delamination growth is needed. Through this procedure, the crack growth rate curves were derived, showing that the rGO reduced the crack propagation distance by 26%, while the HDPlas filler achieved an even greater improvement of 44% compared to the neat composite. SEM observations confirmed that the improved fracture performance is associated with crack bifurcation and nanoparticle pull-out. Notably, the HDPlas nanofiller system showed more extensive pull-out, which provided additional energy dissipation and resulted in superior fatigue performance compared to the rGO nanofiller. Overall, the study highlights that the addition of graphene nanospecies seems to contribute positively to delamination growth resistance in composite materials. Tailoring the type and interaction of nanofiller with the matrix can significantly improve the damage tolerance and long-term reliability of advanced composite structures, especially for applications such as aerospace, automotives and wind energy.

## Figures and Tables

**Table 1:** Initiation and total values of energy release rate for the laminates.

Laminate	G_I,init_ (kJ/m^2^)	Increase (%)	G_I_ (kJ/m^2^)	Increase (%)
Neat	0.24 ± 0.02	-	0.42 ± 0.03	-
rGO	0.30 ± 0.05	25	0.57 ± 0.07	36
HDPlas	0.32 ± 0.07	33	0.70 ± 0.09	67

**Table 2:** Average fatigue onset lives and threshold energy release rate values for the manufactured composites.

	N1%		N5%		G_Ith_ (kJ/m^2^)
Laminate	G-Level 50%	G-Level 80%	G-Level 50%	G-Level 80%	
Neat	112	214	7728	5419	0.0916
rGO	449	400	15,440	7199	0.1134
HDPlas	319	598	39,184	21,536	0.1527

## Data Availability

The original contributions presented in this study are included in the article. Further inquiries can be directed to the corresponding author.

## Abbreviations

The following abbreviations are used in this manuscript:

CFRP	Carbon Fiber-Reinforced Polymer
rGO	Reduced Graphene Oxide
GNP	Graphene NanoPlatelets
DCB	Double Cantilever Beam
UD	UniDirectional
PTFE	PolyTetraFluoroEthylene
CC	Compliance Calibration
SEM	Scanning Electron Microscopy
ENF	End-Notched Flexure
ASTM	American Society for Testing and Materials
